# Computational Methods for Single-Cell Imaging and Omics Data Integration

**DOI:** 10.3389/fmolb.2021.768106

**Published:** 2022-01-17

**Authors:** Ebony Rose Watson, Atefeh Taherian Fard, Jessica Cara Mar

**Affiliations:** Australian Institute for Bioengineering and Nanotechnology, The University of Queensland, Brisbane, QLD, Australia

**Keywords:** single cell imaging, single cell omics, data integration, machine learning, ageing

## Abstract

Integrating single cell omics and single cell imaging allows for a more effective characterisation of the underlying mechanisms that drive a phenotype at the tissue level, creating a comprehensive profile at the cellular level. Although the use of imaging data is well established in biomedical research, its primary application has been to observe phenotypes at the tissue or organ level, often using medical imaging techniques such as MRI, CT, and PET. These imaging technologies complement omics-based data in biomedical research because they are helpful for identifying associations between genotype and phenotype, along with functional changes occurring at the tissue level. Single cell imaging can act as an intermediary between these levels. Meanwhile new technologies continue to arrive that can be used to interrogate the genome of single cells and its related omics datasets. As these two areas, single cell imaging and single cell omics, each advance independently with the development of novel techniques, the opportunity to integrate these data types becomes more and more attractive. This review outlines some of the technologies and methods currently available for generating, processing, and analysing single-cell omics- and imaging data, and how they could be integrated to further our understanding of complex biological phenomena like ageing. We include an emphasis on machine learning algorithms because of their ability to identify complex patterns in large multidimensional data.

## Introduction

Advances in high-throughput techniques have made it possible to collect largescale data from different types of regulatory information that controls a single cell. As a result, modelling approaches that combine multiple layers of cellular information deliver more informative insights than their single mode counterparts (Zhu et al., 2020). This review provides a comprehensive overview of the advanced technologies used for single cell imaging and omics sequencing, and the opportunities that exist to integrate these two types of data. We describe key advances in technologies and outline the major steps that are important for working with these two data types. Case studies are presented to illustrate some examples of integrating imaging and omics-level data. We emphasise the utility of this type of integration by focusing on studies that feature heterogeneous phenotypes in human health like ageing.

The substantial uptake of single cell-based technologies has been attractive in biomedical research because it is a known fact that human phenotypes are heterogeneous. Single cell omics methods like RNA-sequencing (scRNA-seq) have helped resolve this heterogeneity by providing a clearer resolution of data so that pathways and master regulators can be identified with cell type-level specificity (Efremova and Teichmann, 2020). Single cell imaging methods like fluorescence microscopy have made it possible to acquire cellular features like morphology or cell area at such a high-throughput level that quantitative analyses can be done on populations of cells to investigate this heterogeneity (Marklein et al., 2018). Integrating these two types of technologies offers even more substantial benefits for inferring a more comprehensive model of cellular regulation. However, data integration brings additional challenges and single cell imaging and omics-level data have their own computational issues which is a focus of this review.

One of the major barriers to adopting high-throughput single-cell imaging lies with its computational requirements. For example, image processing, analysis and storage of the massive amount of data that is acquired during a simple imaging experiment are not trivial (Swedlow et al., 2021). For a typical research lab, this will usually require additional resources. These issues are compounded when integrating datasets collected from imaging and omics assays, which can drive the dimensions of the dataset into the hundreds of thousands, even whilst the number of biological samples remains small (Mirza et al., 2019). However, solutions are increasingly becoming more available and accessible through high-performance computing options on cloud platforms, along with high quality, open-source image processing and analysis software, and more efficient pipelines.

With the appropriate experimental assay and imaging technology, high-throughput cellular imaging can collect an impressive range of quantitative metrics that describe a single cell (Bray et al., 2016a). From quantifying basic morphological, intensity and textural features, to identifying the structure, number, and spatial distribution of sub-cellular elements such as organelles, proteins, and RNA sequences. Unlike omics technologies, in imaging many of these cellular features can even be measured in the same cell multiple times, giving insight into the spatiotemporal dynamics of single cells without having to destroy the cell (Nozaki et al., 2017). Single-cell imaging can also be used to explore the cause and effect relationship between specific genetic, chemical, and environmental perturbations and a variety of cellular phenotypes (Mattiazzi Usaj et al., 2016). Consequently, microscopy remains the most informative tool for capturing associations and interactions between multiple molecular and cellular elements at high resolution.

Although the use of imaging data is well established in biomedical research, it has primarily been applied to observing phenotypes at the tissue or organ level, often using medical imaging techniques such as MRI, CT and PET (Shen et al., 2017). Such imaging has been complementary to omics-based data in biomedical research, where the goal is to identify associations between genotype and phenotype, along with functional changes at the tissue level (Antonelli et al., 2019). Now that omics and imaging techniques are becoming more accessible, it is feasible that single-cell imaging can act as an intermediary between these levels of information. As a result, integrating single-cell omics and single-cell imaging allows for a more effective and comprehensive characterisation of the underlying mechanisms of a cellular phenotype.

All living organisms experience ageing, a phenomenon that is broadly defined as a gradual decline in physiological integrity, and consequently function, over the lifetime of an organism (López-Otín et al., 2013a). For humans, ageing can manifest through different symptoms, affecting a variety of organs and tissue types in a heterogeneous manner. Despite decades of research into practical and effective ageing interventions, advanced age remains the primary risk factor for many serious and chronic morbidities, including metabolic, cardiovascular, neoplastic, and neurodegenerative disorders (Niccoli and Partridge, 2012). From one individual to another, these age-associated pathologies vary in their severity and onset. Similarly, ageing within an individual is highly heterogeneous, with different tissues, cells and even cellular components that age according to different trajectories and rates.

Ageing is defined by a set of traits, termed the hallmarks of ageing (López-Otín et al., 2013b), which represent the key molecular and cellular components that are affected as organisms age. Once the level of damage within a cell reaches a certain threshold, it can initiate a cellular stress response known as senescence (Bhatia-Dey et al., 2016). Senescent cells secrete a variety of cytokines, chemokines, proteases, and other molecules that drive chronic inflammation in the tissue environment, leading to dysfunction and degradation that manifests as age-associated disease (Childs et al., 2015). Single-cell omics technologies have begun to provide insights into the mechanisms underlying senescence, sources of heterogeneity and the biological ageing process (Uyar et al., 2020). However, a complete picture cannot be formed without the addition of another technology: high-throughput cellular microscopy.

This review outlines the key methods currently available for the processing and analysis of single-cell omics and imaging data. We discuss how these data types can be used to further our understanding of biological processes, with a focus on applications in ageing. An emphasis has been included on machine learning algorithms, which can exceed human abilities in their capacity to identify extremely complex, subtle, and even sub-visual patterns in large multidimensional data. A range of post-hoc analysis methods can then be applied to extract meaningful biological information from these algorithms. We also explore how the integration of single-cell omics and single-cell imaging data using specific machine learning methods can exploit the distinct strengths of each technology to form a comprehensive understanding of ageing at the single-cell level.

## An Overview of Single-Cell Omics Technologies

Historically, the sequencing methods that were used to capture genome-wide information required starting material that exceeded the amounts obtainable from a single cell. As a result, genomics and all of its related -omics technologies, have grown up in an era where information about the activity of genes and pathways has been obtained from mixtures of cells or what is commonly referred to now as “bulk” samples. Measurements obtained from bulk samples result in the loss of cell-specific information because information from individual cells were averaged together to give a single, final data point. The transition from bulk to single cell-based approaches has had a major impact on genomics because it means that differences between cells can now be resolved rather than ignored as before. What used to be considered heterogeneity in data can now be clarified and sourced to differences in cell type or cell state because omics data can be captured for individual cells. The recent advances that have made single cell sequencing possible include improvements in single cell isolation, genome amplification, and barcoding which collectively have provided a platform to source information from different cellular and molecular levels without having to pool starting material. The current goal for genomics and its related technologies is to convert this information into actionable inferences that help describe the underlying biological mechanisms of different cells and tissue types.

Single cell-level omics data has also forced us to consider new implications, constraints and issues for the statistics that must be addressed for the analysis of this data. Because some of these statistical considerations are distinct from their counterparts for bulk data, it is necessary to adapt or invent new quantitative approaches that are appropriate for single cell data. For example, the most popular statistical approach for identifying differentially expressed genes for RNA-sequencing data is typically through an exact test for counts that have been fitted to a negative binomial distribution. Single cell RNA-sequencing data is more complex, with increased zeros and sometimes a multimodal distribution, and differential expression is typically assessed using a Wilcoxon signed-rank test (WSRT). The different statistic is necessary because of the increased heterogeneity in single cell data than in bulk data where the latter can be modelled more reliably with an approach that is based on parametric assumptions.

There are many other tasks in single cell data where the differences in the statistical approaches vary substantially from their bulk data counterparts. One prominent example is the data pre-processing and quality control pipelines where for single cell RNA-sequencing data, identifying low quality cells or detection of doublets are necessary for improving downstream data quality. Another example is clustering single cell RNA-sequencing data into groups of cells with similar expression profiles where the end goal is to identify cell types. This specific task has no direct parallel in bulk data because it is only at the level of single cells that information on cell types can be quantified.

### Single-Cell Genomics

Single-cell genomics commonly refers to the capture of the DNA sequence of all genes in the genome of a single cell. In addition to identifying the genotype of a single cell’s genes, this information enables the detection of rare and unique genomic alterations like single nucleotide polymorphisms (SNPs) and copy number variation (CNV). Understanding what genetic or genomic changes that occur in individual cells is instrumental to early detection of a disease such as in the case of an early-stage embryo with a genetic condition or identifying the spectrum of clonal variation present in a tumour. For most genetic analysis studies, having access to an adequate quantity of high quality DNA is critical. There are various methods for amplifying the genome in preparation for single-cell whole genome sequencing (scWGS), for different applications including single SNP and CNV analysis. For example, multiple displacement amplification (MDA) method (Dean et al., 2002), can be carried out directly from biological samples and provides amplified DNA fragments that are uniformly represented across the genome. This method leverages the φ29 DNA polymerase and random exonuclease-resistant primers in a simple isothermal reaction to amplify DNA strands with >10 kb in length. Similarly, MALBAC (Multiple Annealing and Looping Based Amplification Cycles) (Zong et al., 2012) provides amplified DNA through a series of temperature cycles, starting with melting genomic DNA into a single strand, random annealing of MALBAC primers to the DNA fragment, followed by extension to a semi- and then a full-amplicon. MALBAC claims to have a lower amplification bias as compared to methods with nonlinear amplification techniques like MDA and PCR-based methods. PicoPLEX (Rubicon Genomics PicoPLEX Kit) is a commercially-available whole genome amplification technology that performs DNA amplification of a single cell in a three-step single-tube reaction. Similar to the MALBAC method, the DNA template is denatured and pre-amplified using a quasi-random priming approach, creating a library of hairpin molecules that can be directly amplified into bulk quantities of DNA for further analysis (Table 1).

**TABLE 1:** Single cell multi-omics technologies.

Single cell whole genome sequencing (scWGS)	
Multiple displacement amplification (MDA)	Dean et al. (2002)
Multiple Annealing and Looping Based Amplification Cycles (MALBAC)	Zong et al. (2012)
Rubicon Genomics PicoPLEX Kit	(Rubicon/Takara)
Single cell chromatin accessibility	
scDNase-seq (single-cell DNase sequencing)	Jin et al. (2015)
sci-ATAC-seq (single-cell combinatorial indexing assay for Transposase-accessible chromatin with sequencing)	Cusanovich et al. (2015)
scATAC-seq (single-cell assay for transposase-accessible chromatin using sequencing)	Buenrostro et al. (2015b)
NOMe-seq (nucleosome occupancy and methylation sequencing)	Kelly et al. (2012)
scMNase-seq (single-cell micrococcal nuclease sequencing)	Lai et al. (2018)
Single-cell epigenomics	
scRRBS (single-cell reduced representative bisulfite sequencing)	Guo et al. (2013)
scWGBS (single-cell whole-genome bisulfate sequencing)	Farlik et al. (2015)
snmC-seq (single-nucleus methylcytosine sequencing)	Luo et al. (2017)
sci-MET (single-cell combinatorial indexing for methylation)	Mulqueen et al. (2018)
Single-cell transcriptomic	
Drop-seq	Macosko et al. (2015)
Smart-seq/2 (switching mechanism at 5′ end of the RNA transcript)	Goetz and Trimarchi, (2012); Picelli et al. (2014)
CEL-seq (Cell expression by linear amplification and sequencing)	Hashimshony et al. (2012)
MARS-seq	Jaitin et al. (2014)
STRT-seq	Islam et al. (2011)
Quqartz-seq	Sasagawa et al. (2013)
SPLIT-seq	Rosenberg et al. (2018)
Single-cell proteomics	
MagRC (magnetic ranking cytometry)	Poudineh et al. (2017)
microengraving and droplet microfluidics	Haidas et al. (2019)
PLA (proximity ligation assay) and PEA (proximity extension assay)	Söderberg et al. (2006), Darmanis et al. (2016)
CyTOF (cytometry by time of flight)	Bandura et al. (2009)
SCBCs (Single-cell barcode chips)	Labib and Kelley, (2020)
SCoPE-MS (Single Cell ProtEomics by Mass Spectrometry)	Budnik et al. (2018); He et al. (2020)
Single-cell metabolomics	
ESI-MS (electrospray ionization mass spectrometry)	Huang et al., (2020); Li et al. (2020)
MALDI-MS (matrix-assisted laser desorption/ionization mass spectrometry)	Shanta et al., (2020)
SIMS (secondary ion mass spectrometry)	Leo et al., (2019)
Transcriptome with genome	
DR-seq (gDNA-mRNA sequencing)	Dey et al. (2015)
G&T-seq (genome and transcriptome sequencing)	Macaulay et al. (2015)
SIDR (simultaneous isolation of genomic DNA and total RNA)	Han et al. (2018)
TARGET-seq	Rodriguez-Meira et al. (2019)
scTrio-seq (single-cell triple omics sequencing	Hou et al. (2016)
Transcriptome with epigenome	
sci-CAR (single-cell combinatorial indexing chromatin accessibility and mRNA)	Cao et al. (2018)
SNARE-seq (single-nucleus chromatin accessibility and mRNA expression sequencing)	Chen S. et al. (2019)
scNMT-seq (single-cell methylation and transcription sequencing)	Clark et al. (2018)
scM&T-seq (single-cell methylome and transcriptome sequencing )	Angermueller et al. (2016a)
scTrio-seq	Hou et al. (2016)
Transcriptome with proteome	
PEA/STA (proximity extension assay/specific RNA target amplification)	Genshaft et al. (2016)
PLAYR (proximity ligation assay for RNA)	Frei et al. (2016)
CITE-seq (cellular indexing of transcriptomes and epitopes by sequencing)	Stoeckius et al. (2017)
REAP-seq (RNA expression and protein sequencing assay)	Peterson et al. (2017)
RAID (single-cell RNA and immunodetection)	Gerlach et al. (2019)
ECCITE-seq (CRISPR-compatible cellular indexing of transcriptomes and epitopes by sequencing)	Mimitou et al. (2019)

Single-cell genomics coupled with other single cell technologies can be used to construct information about the genome in a functional capacity and infer what molecular mechanisms underlie biological phenomena like cancer and development. For example, single-cell genomics has been extensively used in cancer to identify carcinogenic driver mutations, understand intratumor heterogeneity and its consequence on the transcriptome (Lim et al., 2020). In developmental biology, single-cell genome sequencing has been instrumental for reconstructing cellular ancestries in the form of a lineage tree (Hu et al., 2018a).

Numerous studies have used multi-omics sequencing to make the link between regulation of the genome with other omics at a single cell level (Lee et al., 2020). For example, Dey et al. (2015) used simultaneous sequencing of genomic DNA and mRNA from a single cell to investigate the correlation of CNVs to variability of the transcriptome in individual cells. They found that variations in CNV could potentially drive the gene expression variability observed in single cells.

### Single-Cell Epigenomics

The epigenome is defined as the set of all changes occurring in a genome that does not involve alterations in DNA. Studying the epigenome therefore involves many different types of data depending on the specific epigenetic modification. For instance, a common type of epigenetic modification is DNA methylation where the addition of a methyl group to cytosine is a regulatory mechanism for controlling gene expression. Adaptations to bulk-level assays for capturing genome-wide DNA methylation events have resulted in the availability of single cell-level approaches to sequencing single cell DNA methylomes (Table 1).

Single-cell epigenomics sequencing provides insights into how the epigenome affects the transcriptome in a cell. There are several single-cell epigenomics sequencing technologies that provide information on DNA modifications, DNA accessibility and chromosome conformation. For example, ATAC-seq (Assay of Transposase Accessible Chromatin sequencing) is an assay designed for detecting chromatin accessibility. In this method hyperactive Tn5 transposases, loaded with sequencing adaptors, are probed in regions of open chromatin (*i.e.* nucleosome-free regions) and are used to generate sequencing libraries that can be amplified and sequenced (Buenrostro et al., 2015a). To capture *cis*-regulatory elements in individual cells, conventional ATAC-seq techniques have been implemented on droplet-based platforms for massively parallel sequencing and mapping transposase-accessible chromatin in tens of thousands of single cells (Yan et al., 2020). Other techniques include bisulfite sequencing that measures DNA methylation, Hi-C sequencing for measuring chromatin accessibility and chromosome conformation, and chromatin immune-precipitation that measures histone modifications and protein-DNA interaction (Lee et al., 2020).

### Single-Cell Transcriptomics

The transcriptome is the set of all RNA transcripts, including coding (messenger RNA) and non-coding (such as microRNA and long non-coding RNA) which deliver information about protein-coding genes or RNA regulatory tasks, respectively. Single-cell transcriptomic technologies capture the gene expression levels of the transcriptome from thousands of single cells simultaneously (Hériché et al., 2019). The development of high-throughput protocols for single cell isolation and cell-specific barcoding technologies has enabled the generation of these datasets that allow cell-to-cell heterogeneity to be studied in a cellular population. Single-cell transcriptomic technologies have led to a host of new discoveries, including the detection of rare and new cell subtypes, the capture of cellular heterogeneity within a tissue, the identification of cellular states, and creating maps of developmental trajectories of specific cell types through pseudo temporal modelling and trajectory inference (Table 1).

Single cell methods differ in the strategies they adopt for individual protocol steps such as single cell isolation, library contraction and sequencing design as they are developed for different purposes. For example, Quartz-seq, MARS-seq and CEL-seq are UMI-based methods that measure transcripts at 3’ end whereas Smart-seq and Smart-seq2 measure the full-length transcript (Ziegenhain et al., 2017; Lee et al., 2020). CEL-seq and Smart-seq use Fluidigm C1 (Wang and Navin, 2015) single cell isolation method while MARS-seq and Smart-seq2 use a FACS technique (Wang and Navin, 2015; Ziegenhain et al., 2017) (Table 1). Several largescale projects have been initiated to catalogue the comprehensive set of cell types in the human body (e.g. the Human Cell Atlas project) and to identify the spectrum of cell states at different stages of life (He et al., 2020; Lee et al., 2020).

### Single-Cell Proteomics

Single-cell proteomics is one of the more recent areas of growth and new technologies to understand proteins at the single level and at scale are beginning to emerge. This is because unlike DNA and mRNA, proteins cannot be amplified. Nevertheless, there are several technologies that are mainly based on the applications of fluorescence-activated cell sorting (FACS), Western blotting, metal-tagged antibodies followed by mass cytometry to sort, qualify phenotypes and high-multiplexing protein analysis (He et al., 2020). These methods are able to capture and analyse cell surface, cytoplasmic and secreted proteins (Labib and Kelley, 2020). For example, magnetic ranking cytometry (MagRC) (Poudineh et al., 2017) detects cell-surface proteins, while microengraving and droplet microfluidics (Haidas et al., 2019) detect the secreted protein. For cytoplasmic protein detection, methods include single-cell western blotting, proximity ligation assay (PLA) (Söderberg et al., 2006), proximity extension assay (PEA). Methods such as flow cytometry and single-cell barcode chips (SCBCs) are used for the analysis of proteins at all three cellular locations (Labib and Kelley, 2020). Although methods for single cell proteomics are mainly based on a limited number of proteins, the recently developed Single Cell ProtEomics by Mass Spectrometry (SCoPE-MS) technique is able to detect more than 1000 proteins in a single cell (He et al., 2020; Budnik et al., 2018). It is worth highlighting that although innovations in mass spectrometry (MS) have improved the scope and scale of these technologies, as in the case of cytometry by time of flight (CyTOF (Bandura et al., 2009)), these methods are still not comparable to omics-level throughput.

### Single-Cell Metabolomics

The aim of single-cell metabolite profiling is to study the effect of small molecules and metabolites in an epigenetic and transcriptomic profile in a single cell. Metabolites are arguably the end product of the basic central dogma process performed in the cell, providing a more immediate and holistic insight about the cellular phenotype. Metabolomics inform about the exact downstream effect and ultimate fates of the analytes, an information that other omics technologies fail to generate (Minakshi et al., 2019).

Screening single-cell metabolite profiles is challenging because these biomolecules have relatively short lifespans, are structurally diverse and chemically unstable *in vitro* (Minakshi et al., 2019; Zhu et al., 2021). However, refinements in the current single cell isolation techniques, mass spectrometry (MS) and high-throughput microfluidic-based methods have led to the detection of a limited number of metabolites present in the cell (Comi et al., 2017; Zhang and Vertes, 2018; Duncan et al., 2019; He et al., 2020). These methods include electrospray ionization mass spectrometry (ESI-MS) (Huang et al., 2020; Li et al., 2020), matrix-assisted laser desorption/ionization mass spectrometry (MALDI-MS) (Shanta et al., 2020)and secondary ion mass spectrometry (SIMS) (Leo et al., 2019). Coupled with separation-based methods, MS is the most sensitive method for detecting a wide range of metabolites in a single cell. After the single cell is lysed, the complete cellular metabolome is separated by chromatography or electrophoresis on an automated platform such as a microfluidic device. The separated metabolites are then delivered to a MS platform for metabolite identification, quantitation, or downstream analysis (Minakshi et al., 2019). For a comprehensive review on single cell isolation strategies, sample preparation methods and single-cell metabolomics technologies refer to (Minakshi et al., 2019; Feng et al., 2020; Dueñas et al., 2021; Zhu et al., 2021).

## Integrative Multi-Omics Sequencing Technologies

### Transcriptome With Genome

An important advantage of detecting multiple molecules from a single cell is that genotype-phenotype correlations can be accurately identified. These paired approaches can be used to link variation in genotype with their corresponding variation in transcriptional responses, and this information can be expanded to further applications like constructing lineage trees that map this variation. Detecting genomic mutations can also be performed with greater accuracy because they can be verified with corresponding mutations occurring in the RNA. Several methods have been developed for the simultaneous extraction and sequencing of the genome and transcriptome of a single cell (Table 1). These technologies differ in terms of how they capture cytoplasmic mRNA and nuclear DNA (genomic DNA). For example, scTrio-seq requires the cytoplasm and nucleus to be physically separated by centrifugation whereas G&T-seq separates poly-A-tailed mRNAs from gDNA using oligo-dT-coated magnetic beads. Next, the mRNA and gDNA will be independently amplified and sequenced using single-cell mono-omics sequencing technologies such as PicoPLEX (for gDNA) and Smart-seq2 (for mRNA). For further details, the characteristics of these technologies are summarised in (Hu et al., 2018b) and (Lee et al., 2020).

### Transcriptome With Epigenome

Changes in DNA methylation and chromatin accessibility are directly linked to the regulation of gene expression. Advances in single-cell epigenomics and transcriptomics have now made it feasible to study how DNA methylation and histone modification vary with changes in transcription in a single cell (Clark et al., 2016). scM&T-seq (single-cell methylome and transcriptome sequencing) (Angermueller et al., 2016a) was the first method to be reported for combined DNA methylome and transcriptome analysis. Since then, other methods that combine the transcriptome with the epigenome have been developed, including scTrio-seq (Hou et al., 2016) which allows for the simultaneous profiling of DNA, methylome, genome and transcriptome within a single cell. A variety of methods exist where they differ in terms of the approaches that they adopt for isolating DNA and RNA and the subsequent mono-omics sequencing technology employed (Hu et al., 2018b; Lo and Zhou, 2018; Lee et al., 2020) (Table 1).

### Transcriptome With Proteome

Methods that measure the transcriptome and proteome of a single-cell (Table 1) are designed for capturing proteins at different cellular locations and throughputs. For example, CITE-seq and REASP-seq can quantify cell-surface proteins with more than 80 antibodies and detect more than 20,000 genes in a single workflow (Hu et al., 2018b). RAID-seq on the other hand detects intracellular or phosphorylated proteins together with mRNA expression. ECCITE-seq is an extension of the CITE-seq method which provides a range of multi-modal information including transcriptome, protein, clonotype, and CRISPR perturbation data at the single cell level (Mimitou et al., 2019; Lee et al., 2020). While the scale of single cell proteomics approaches is increasing with more modern innovations, it is important to recognize that the expectations for the throughput of these single-cell proteomics and integrated transcriptomic-proteomic approaches are not the same as for single-cell transcriptomic or epigenomic methods. At this stage, being able to capture single-cell level data for proteins is still only for smaller numbers of molecules at a time.

## Methods for Multi-Omics Data Analysis

All single-cell omics data are usually subjected to a variety of pre-processing steps that include alignment back to a reference, filtering to remove noise, and evaluation of quality control steps to assess overall reliability of the data. Subsequently, the data is subjected to a normalisation step which aims to reduce the amount of technical variation and thus increase the signal-to-noise ratio in the data. Other considerations for pre-processing of single cell data include detecting datapoints that may correspond to more than one cell, referred to as a doublet, and removing them from further analysis. Batch effects may induce patterns in the data that distract from studying genuine biological effects. The removal of these batch effects through different correction methods is therefore an important pre-processing step for this data type. Different statistical methods have been developed to address these pre-processing goals that are specific for their respective data type.

The applications of methods for sc-RNA data analysis have begun to evolve into a predictable workflow. These analysis steps include cell type identification from a heterogeneous cell population, regulatory-network based inference to identify regulatory relationship among marker genes, and cellular trajectory inference to study the temporal dynamics of the transcriptome during development or where cells may adopt one state along a continuum as they transition between states (Hwang et al., 2018; Lee et al., 2020).

Cell type identification from scRNA-seq data is mainly based on clustering methods (*e.g.* k-means, hierarchical, and graph-based) that operate off data that has been subjected to a dimensionality reduction (DR) technique. Principal component analysis (PCA) is a well-established unsupervised linear DR method. Other commonly used approaches are non-linear DR methods including t-distributed stochastic neighbour embedding (t-SNE) (van der Maaten and Hinton, 2008), locally linear embedding (LLE) (Roweis and Saul, 2000; Tenenbaum et al., 2000) and deep count autoencoder (DCA) (Eraslan et al., 2019). Among the frequently used packages for clustering and cell type annotation are Seurat (Stuart et al., 2019), SNN-cliq (Xu and Su, 2015), Garnett (Pliner et al., 2019) and SingleR (Aran et al., 2019). For an extensive review on cell type annotation and clustering methods refer to (Abdelaal et al., 2019; Wu and Zhang, 2020).

Cell trajectory inference involves ordering cells based on their transcription profile to identify continuous cell states and branch points that represent key fate decisions along the trajectory. There is a plethora of trajectory inference packages with each relying on a different method and trajectory type (Saelens et al., 2019). For example, Monocle (Qiu et al., 2017) and SlingShot use a tree-based method (Street et al., 2018), PAGA (Wolf et al., 2019) uses a graph-based method, Wishbone (Setty et al., 2016) uses a bifurcation method whereas GPfates (Lönnberg et al., 2017) is based on a multifurcation method.

Gene regulatory networks are important models for understanding the gene-gene and other types of interactions that control the transition from one cell type to another (Pratapa et al., 2020). Among the commonly used network-based inference methods that have been developed specifically for scRNA-seq data, some of the popular ones include the SCNS toolkit (Moignard et al., 2015), SCODE (Matsumoto et al., 2017) and SCENIC (Aibar et al., 2017).

Methods for single cell genomics and epigenomics analysis allow for the identification of genetic aberrations and epigenetic changes occurring at the single cell level (Gawad et al., 2016; Lee et al., 2020). Methods for identifying CNVs from scWGS data include Ginkgo (Garvin et al., 2015), baseqCNV (Fu et al., 2019), SCNV (Wang et al., 2018), SCCNV (Zhang et al., 2019), and SCOPE (Wang et al., 2019a). Moreover, several methods have been developed for the effective identification of SNVs from single cell whole genome sequencing data such as SCcaller (Dong et al., 2017), baseqSNV (Fu et al., 2019), MonoVar (Zafar et al., 2016), and SCAN-SNV (Luquette et al., 2019). Methods for identifying open chromatin sites and peak identification include chromVAR (Schep et al., 2017) and SCALE (Xiong et al., 2019), respectively. For an extensive review on these and other methods on multi-omics data analysis, we refer readers to (Hu et al., 2018b; Hwang et al., 2018; Chen H. et al., 2019; Saelens et al., 2019; Lee et al., 2020; Pratapa et al., 2020; Wu and Zhang, 2020).

## Single-Cell Imaging

The growing need to visualise cellular elements at a molecular scale has driven rapid developments in all facets of microscopy imaging (Galler et al., 2014). Advances in single cell imaging have now gone beyond just visualising cells. Instead, identification and quantification of cellular and sub-cellular elements are routine. A variety of technologies have been developed or adapted to capture spatial, temporal, and morphological information at the single-cell sub-cellular level. For example, the spatial distribution of hundreds to thousands of unlabelled molecular species can be visualised at sub-cellular resolution with Imaging Mass Spectrometry (Buchberger et al., 2018). Cryo-electron microscopy has undergone a “resolution revolution,” where it is now capable of single-particle imaging at resolutions quickly approaching the sub-nanometre scale (Danev et al., 2019). Several imaging modes of atomic force microscopy have been developed to offer nanometre resolution imaging of structures in live cells, whilst simultaneously characterising mechanical, kinetic, thermodynamic and electrostatic properties (Dufrêne et al., 2017). Despite the rapid expansion of such sophisticated instruments and technologies, optical microscopy has remained one of the foremost approaches in single-cell imaging, and as such will be the focus of this review.

### Optical Microscopy

Optical microscopy has played a foundational role in the discovery and characterisation of biological structures, molecules, and processes since the 17th century. This type of technology remains popular due its simplicity, flexibility, and non-invasive nature (Masters, 2008). Although the core concept of utilising a light source and one or a series of lenses to generate magnified images remains, advances in optical and mechanical components have transformed the quality and functionality of optical microscopes considerably. Most notably, the automation of the sample preparation and image acquisition processes such as liquid handling, focusing, sample positioning and illumination and detection multiplexing, have transformed optical microscopes into sophisticated systems that are capable of imaging thousands to hundreds of thousands of samples at a single-cell resolution in a matter of hours (Lock and Strömblad, 2010; Mattiazzi Usaj et al., 2016; Mikami et al., 2018). These developments have also led to the incorporation of optical microscopes into other high-throughput single-cell technologies, as with imaging flow cytometers, enabling the collection of additional information on morphological, spatial, and textural features (Stavrakis et al., 2019). The quantity and diversity of cellular structures and biomolecules that can be specifically and sensitively identified within a single cell has also advanced significantly (Ozawa et al., 2013). These developments have enabled the systematic and quantitative investigation of single-cell biology with imaging data at similar scale and accessibility previously only seen in sequencing technologies, but with significant spatial and temporal information (Wollman and Stuurman, 2007).

These high-throughput microscopy systems and sophisticated labelling technologies can also be paired with large-scale systematic perturbations to provide insights into the influence of genetic or environmental factors on various cellular attributes (Boutros et al., 2015; Pegoraro and Misteli, 2017). Screening of comprehensive small molecule libraries is a common strategy for rapidly identifying and validating compounds in drug discovery and development (Bray et al., 2017; Boyd et al., 2020). Alternatively, chemical-genetic screens use libraries of characterised compounds, where the resulting phenotype (forward screening) or biological target (reverse screening) are known in advance (Choi et al., 2014). These screens facilitate the discovery of specific genes, proteins or pathways involved in cellular phenotypes of interest (Pegoraro and Misteli, 2017). Similarly, genetic screens utilise gene perturbation technologies such as RNAi (Schmidt et al., 2013) and CRISPR/Cas9 (Rauscher et al., 2017) to enable knockout, knockdown, or overexpression studies to target tens of thousands of genes at a time (Schuster et al., 2019).

### Fluorescence Microscopy

In imaging, an investigation into complex aspects of cellular biology often starts with labelling for specific identification. Depending on the study, a variety of biological attributes can be labelled, including certain cellular structures, organelles, macromolecules or even processes of interest. Fluorescence microscopy is an approach that offers excellent labelling specificity through the use of molecules called fluorophores, which have the capacity to absorb light of a specific wavelength and subsequently re-emit it at a longer wavelength. Paired with the properties of the fluorescence microscopes, high detection sensitivity can be achieved with minimal cell perturbation (Shashkova and Leake, 2017). There is also an increasing variety of fluorescence microscopy techniques available to suit a diverse range of applications where each come with their own trade-offs (Jensen, 2012; Combs and Shroff, 2017).

For example, confocal fluorescence (CFM) and light-sheet fluorescence (LSFM) microscopy are two techniques capable of producing high-resolution imaging of focal planes deep within samples, known as optical sectioning. This enables the reconstruction of three-dimensional cellular or subcellular structures in specimens, providing valuable spatial information (Long et al., 2012). Optical sectioning in CFM is achieved through the use of point-like illumination and detection pinholes that reject out-of-focus light. Whilst being highly cost-effective and accessible, CFM image-acquisition is slow, and produces moderate photo-bleaching and toxicity, as light must pass through the sample to reach the plane of interest (Jonkman and Brown, 2015). In comparison, LSFM performs high-speed optical sectioning by projecting a thin light sheet onto the sample from the side. This restricts illumination to the focal plane of interest, reducing photo-bleaching and toxicity significantly (Zagato et al., 2018). As a result, LSFM can perform high-resolution 3D imaging in live samples for long periods of time. Hof, Moreth (Hof et al., 2021) recently used LSFM to perform live imaging of the dynamic processes of organoid morphogenesis at the single-cell scale for up to 7 days. However, implementation of LSFM is substantially more challenging than CFM, including extensive and non-standard sample preparation (Zagato et al., 2018).

Super-resolution fluorescence microscopy (SRM), or nanoscopy are techniques that have the capacity to surpass the diffraction limit of optical resolution of approximately 200 nm are also available (Schermelleh et al., 2019). Several SRM techniques have the capacity to generate 2D and 3D images at a resolution of <50nm, with some reaching as high as <10 nm. Most SRM methods can also be successfully applied to live-cell imaging, with some approaches demonstrating a temporal resolution of only milliseconds (Balzarotti et al., 2017). SRM has already enabled the observation and quantification of *in situ* protein aggregation associated with various neuro-degenerative diseases, protein mobility within mitochondrial sub-compartments, and even the discovery of entirely new subcellular structures (Balzarotti et al., 2017). Several comprehensive reviews of SRM in cellular biology are available for further information (Sahl et al., 2017; Vangindertael et al., 2018; Schermelleh et al., 2019; Jacquemet et al., 2020).

### Advanced Fluorescence Microscopy Techniques

The modification of fluorescence microscopy approaches has also created advanced techniques for the precise quantification of complex cellular dynamics in real time and at the nano-scale (De Los Santos et al., 2015). Data generated with these methods reveal insights into intra-cellular processes that are difficult to achieve with standard approaches. These techniques are highly tuned to specific applications through exploitation of specific fluorescence properties. For example, Fluorescence recovery after photobleaching (FRAP), Fluorescence Loss In Photobleaching (FLIP) and Fluorescence Localisation after Photobleaching (FLAP) all rely on the photobleaching of fluorophores that occurs due to the reactions between the fluorophore and the surrounding molecules during excitation (Ishikawa-Ankerhold et al., 2012). These techniques are commonly used to investigate molecular motility and diffusion, and explore the connections and molecular exchange happening between cellular compartments (Drummen, 2012).

Förster Resonance Energy Transfer (FRET) techniques are based on the distance-dependent transfer of excitation energy between a donor and an acceptor fluorophore, and can be adapted for an extensive variety of applications, including the motility, localisation, interactions and structural relationships of several molecular species (Algar et al., 2019). The application of this technique can facilitate the characterisation of complex processes such as signalling pathways or protein-folding dynamics (Krainer et al., 2019). Fluorescence Lifetime Imaging Microscopy (FLIM) capitalises on the exponential decay in fluorescence emission after excitation, which is influenced by minute changes in the microenvironment such as pH, temperature or ion concentration (Datta et al., 2020). Many of these techniques provide complementary information, and as such are frequently applied in combination to yield comprehensive and rich imaging datasets of complex biological phenomena.

### Labelling Strategies

Fluorophores commonly take the form of fluorescent proteins (Chudakov et al., 2010), synthetic organic molecules (Terai and Nagano, 2013), and fluorescent nanoparticles (Pratiwi et al., 2019), with assorted physiochemical properties to complement different labelling and microscopy techniques (Nienhaus and Nienhaus, 2017). An ongoing challenge of fluorescence microscopy is the limited capacity for *in situ* label multiplexing due to the broad excitation and emission spectra of many fluorophores, which results in bleed-through of signal between channels during imaging. As a result, only a small number of molecular targets can be imaged simultaneously in the same cell. To overcome this, the synthesis of new fluorescent labels with properties to extend the opportunities for effective multiplexing, such as increasingly narrow emission bands (Martino et al., 2019; Pandey and Bodas, 2020) or advanced optical encoding (Lin et al., 2018; Zhai et al., 2020) is a major area of focus, with fluorescent nanoparticles showing particular promise (Lee et al., 2018).

For both fixed and live cell imaging, preferential labelling can be employed to zoom in on certain cellular locations or types of molecules such as basic proteins, lipids, or nucleic acids. For example, the nucleus of live cells is commonly visualised using Hoechst 33342, a membrane-permeable dye which preferentially binds to AT-rich regions of double-stranded DNA (Chazotte, 2011). Fluorescent labelling of cellular components including membranes, organelles, cytoplasm, cytoskeleton, lysosomes, lipid droplets is similarly possible. Assays based on applying a combination of such stains, such as Cell Painting (Bray et al., 2016b), are popular for the generation of rich morphological profiles of single-cells at scale. Also available are fluorophores that report on particular chemical properties of the cellular environment, such as metal ions (Domaille et al., 2008), pH (Han and Burgess, 2010) or temperature (Okabe et al., 2018), often within specific compartments (Mizukami, 2017). Alternatively, when a certain molecule is of interest, fluorophores may be fused to a biomolecule, such as a protein, peptide, or nucleic acid, which acts as a specific probe for the target molecules. Common examples of this approach include immunofluorescence, Fluorescence *In Situ* Hybridization (FISH) and Genetically-Encoded labelling.

Immunofluorescence labelling uses antibodies with high specificity for a single target, typically a protein, as a probe (Joshi and Yu, 2017). This labelling technique is highly versatile, with an extensive range of commercially available fluorophore-labelled antibodies, which can be applied in different combinations to enable the labelling of several targets in a single cell (Buchwalow et al., 2005). Larger scale label-multiplexing can be achieved via performing cyclic immunofluorescence, whereby multiple rounds of labelling and imaging are conducted through the removal or inactivation of the fluorophore after each round (Wählby et al., 2002; Buchwalow et al., 2005; Ko et al., 2020). However, the applications of immunofluorescence for live cell imaging are generally limited to cell-surface or extra-cellular targets, as cells must be fixed and permeabilised before larger molecules such as antibodies are able to enter (Griffiths and Lucocq, 2014).

FISH techniques use fluorophore-labelled short nucleic acid sequences as the targeted probes of complementary RNA or DNA sequences (Huber et al., 2018). They are commonly applied to study genetic aberrations such as duplications, deletions, insertions, and translocations from the single gene to whole chromosome scale. Single-molecule FISH (smFISH) is a significant variation of FISH that allows for the accurate targeting and detection of individual RNA molecules, providing quantitative information on sub-cellular abundance, localisation and co-localisation of specific RNA sequences (Femino et al., 1998; Raj et al., 2008). smFISH can also be applied to many types of RNA molecules, including messenger RNA (mRNA) (Femino et al., 1998), long non-coding RNAs (Cabili et al., 2015), and ribosomal RNA (Buxbaum et al., 2014).

The smFISH techniques have been expanded further to accommodate greater scale in the number of molecules that can be detected within a single cell. For example, one adaptation called SeqFISH+ was able to capture *in situ* imaging of mRNAs for 10,000 genes in individual cells at high resolution (Eng et al., 2019). Whilst FISH has been traditionally performed in fixed cells, CRISPR live-cell fluorescent *in situ* hybridization (LiveFISH) has recently been developed, enabling real-time imaging of DNA and RNA dynamics in live cells (Wang et al., 2019b).

Genetic encoding of labels, typically through fusion with the gene of a target protein at the DNA level, is a popular technique that ensures excellent target specificity *in vitro* and *in vivo*. GE labels may be intrinsically fluorescent proteins (Thorn, 2017) or tags designed to bind exogenous fluorophores with high specificity (Elia, 2021). Genetically-encoded labelling may also be used to label secondary targets, such as nucleic acids via RNA- or DNA-binding protein domains, or targeted to organelles of interest using specific protein localisation signals (Chudakov et al., 2010). Genetically-encoded sensors are also available for the visualisation and measurement of intra- and extra-cellular physiological, chemical and mechanical properties *in vivo* (Germond et al., 2016; Cost et al., 2019).

**FIGURE 1:**
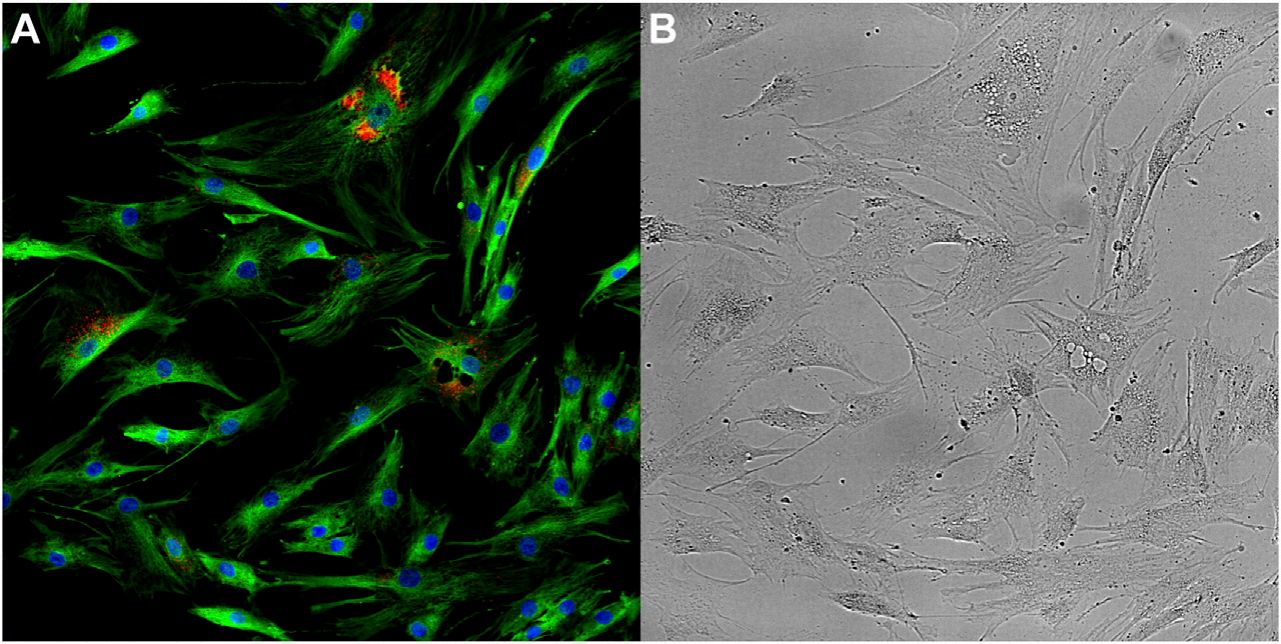
Optical microscopy images taken of ageing mesenchymal stem cells. Fluorescence image **(A)** provides information on the abundance and distribution of DNA (blue), α-Tubulin (green) and Senescence-associated beta-galactosidase (red). Brightfield image **(B)** provides information on the cellular and sub-cellular morphology. Images have been enhanced for visualisation.

Single-cell imaging can also be conducted without the use of fluorescent labelling, using transmitted- or reflected-light microscopes. Label-free microscopy is a valuable technique for the study of cellular biology, offering greater simplicity and lower perturbation than many label-based methods, including fluorescence microscopy (Kasprowicz et al., 2017). Furthermore, label-free imaging techniques typically offer distinct but complementary information to fluorescence microscopy, and as such the two techniques are often applied together (Figure 1). Brightfield microscopy creates a dark image on a light background as light is differentially absorbed, reflected, or refracted by biological structures. Moreover, a variety of techniques, such as darkfield, phase-contrast, polarised light, and differential interference contrast microscopy, have been developed with the capacity to enhance contrast optically, without compromising resolution, and resulting in detailed imaging of subcellular structures (Murphy and Davidson, 2012). The information that can be extracted from label-free images generated with such techniques is also expanding with the development of powerful computational algorithms. For example, a number of *in silico* labelling methods have been developed in recent years, with the capacity to predict multiplexed fluorescent labels in novel, unlabelled images with high accuracy in live and fixed cells (Christiansen et al., 2018). A recent model from Cheng, Fu (Cheng et al., 2021) predicts labels corresponding to the sub-cellular structures DNA, actin, endosome and the Golgi apparatus, as well as labels informing of cellular events such as proliferation and apoptosis. Similar models have been developed with the capacity to predict fluorescence labelling of 3D images (Ounkomol et al., 2018; Guo et al., 2020).

## Methods for Single-Cell Imaging Data Analysis

The development of sophisticated and automated methods for the processing and analysis of imaging data, typically via machine learning (ML) and computer vision (CV), has contributed significantly to the increased popularity of biological image-based research (Danuser, 2011; Meijering, 2020). Despite this, the diversity of assays, techniques, and technologies available for generating cellular imaging data, along with the variability of experimental conditions and lack of standard imaging protocols, precludes the development of a ‘one-size-fits-all’ pipeline (Eliceiri et al., 2012). As such, only a broad overview of common approaches and generalised open source tools for the processing and analysis of fluorescence microscopy image data will be discussed in this review.

Some popular open source tools available for single-cell image data analysis include Icy (de Chaumont et al., 2012), ilastik (Berg et al., 2019), Microscopy Image Browser (Belevich et al., 2016), BioImageXD (Kankaanpää et al., 2012), Cytokit (Czech et al., 2019), KNIME (Fillbrunn et al., 2017), CellProfiler (McQuin et al., 2018) and FIJI (Schindelin et al., 2012) (ImageJ (Abràmoff et al., 2004)). The majority of these tools offer an implementation via a graphical or command line interface, and some in programming languages such as Python (Van Rossum, 2009) or R (R Core Team, 2020). Typically, these tools provide a variety of processing and analysis methods that can be “mixed and matched,” allowing the user to develop a customised pipeline to suit their specific needs. For example, CellProfiler includes over 70 independent modules designed for unique tasks, whilst there are several thousand modules available in the ImageJ ecosystem (McQuin et al., 2018). There are also a variety of powerful image processing libraries available in programming environments, including Scikit-image (van der Walt et al., 2014), Pillow (Clark, 2015) and OpenCV (Bradski, 2000) for Python, and EBImage (Pau et al., 2010), imageHTS (Pau et al., 2020) in R. These, along with a variety of independently developed packages, can be applied in a similar manner for the development of a customised pipeline. Regardless of the nature of the interface, a conventional pipeline for single-cell imaging data consists of three main components: pre-processing for the correction of experimental or imaging artifacts, segmentation of the objects of interest, and an analysis of these objects.

### Pre-Processing

The extent and specific methods applied for pre-processing of an image dataset will vary significantly depending on the type and quality of the images. Typically, all raw biological image data will require some form of denoising (Meiniel et al., 2018). A common source of systematic noise in microscopy imaging data is the presence of non-uniform illumination of the Field Of View (FOV), resulting from factors such as the light source, optical path, camera nonlinearity, or dust and staining artifacts. If left uncorrected, this non-uniformity can bias the measurements of properties of interest such as textural and intensity features, as well as interfere with the quality of processing steps downstream (Dey, 2019). The variety of illumination correction methods available is extensive (Singh et al., 2014; Smith et al., 2015; Peng et al., 2017; Nordenfelt et al., 2018), and are reviewed elsewhere for both general (Piccinini et al., 2012; Dey, 2019) and specific use cases (Liu et al., 2017). Other common pre-processing steps may include deconvolution to correct for signal blurring (Swedlow, 2013) and stitching and registration for samples split over multiple FOVs or imaged in multiple planes, wavelengths or modalities.

### Object Segmentation

The accurate detection and segmentation of individual cells, or sub-cellular regions of interest, is an essential but challenging step in the quantitative analysis of cellular imaging data at the single-cell scale (Meijering, 2012). Traditional approaches to segmentation include thresholding (Otsu, 1979), feature detection (Kass et al., 1987) and watershed-based (Beucher and Meyer, 1993) methods. For particularly heterogeneous, noisy or complex datasets, machine learning models including U-Net (Falk et al., 2019), DeepCell (Van Valen et al., 2016), CDeep3M (Haberl et al., 2018), and CellPose (Stringer et al., 2021), are a popular choice. The segmentation of label-free images can be particularly challenging (Cameron et al., 2020; Liu et al., 2021a), and as such a number of methods have been developed specifically for this task (Vicar et al., 2019). A variety of segmentation methods designed for specific cell types (Li J et al., 2019; Salvi et al., 2019) or datatypes, such as 3D images (Çiçek et al., 2016), are also available. The performance of segmentation methods have been reviewed and compared in detail elsewhere (Dima et al., 2011; Thomas and John, 2017; Caicedo et al., 2019; Cameron et al., 2020).

### Analysis

Analysis of single-cell imaging data relies on extracting informative descriptors of phenotypic characteristics, or *features*, from the images. These features may be manually designed (handcrafted), and selected by the user, or automatically extracted from the data using machine learning algorithms, such as multi-layer artificial neural networks (ANNs). Using handcrafted features is often the more labour intensive approach, however they are also typically easier to interpret, and may even be defined in biologically meaningful terms such as cell membrane circularity or nuclei intensity (Caicedo et al., 2017). Automatically learned features usually take the form of abstract data representations, which are less intuitive, but may also more effectively capture the complexity of heterogenous and high dimensional datasets (Razavian et al., 2014).

These image-derived features describe phenotypic profiles of the system or condition under study (Caicedo et al., 2017; Grys et al., 2017), and are routinely utilised to group cells according to type (Zhang et al., 2017; Yao et al., 2019) or specific processes, such as phases of cell cycle (Eulenberg et al., 2017) via classification or clustering methods. Outlier detection methods can also be applied to identify rare or novel cell-types within heterogeneous populations (Mattiazzi Usaj et al., 2020). Phenotypic profiling of cellular responses to chemical (Kleinstreuer et al., 2014), environmental, and genetic (Rohban et al., 2017) perturbations is frequently applied for functional annotation and classification of the perturbants (Caicedo et al., 2016). Another common analysis is the quantification of the abundance and sub-cellular localisation of proteins (Pärnamaa and Parts, 2017) or RNA molecules (Samacoits et al., 2018) of interest. Other applications include lineage trajectory inference (Buggenthin et al., 2017), which commonly makes use of cell-tracking methods on live, long-term imaging data to accurately ascertain lineage progression (Piltti et al., 2018; Lugagne et al., 2020). Object tracking methods can be similarly applied to study subcellular dynamic processes, such as binding dynamics (Presman et al., 2017) or molecule trafficking (Chen et al., 2016), among others (Nketia et al., 2017; Brandão et al., 2021).

### Multimodal Data Integration Techniques

**FIGURE 2:**
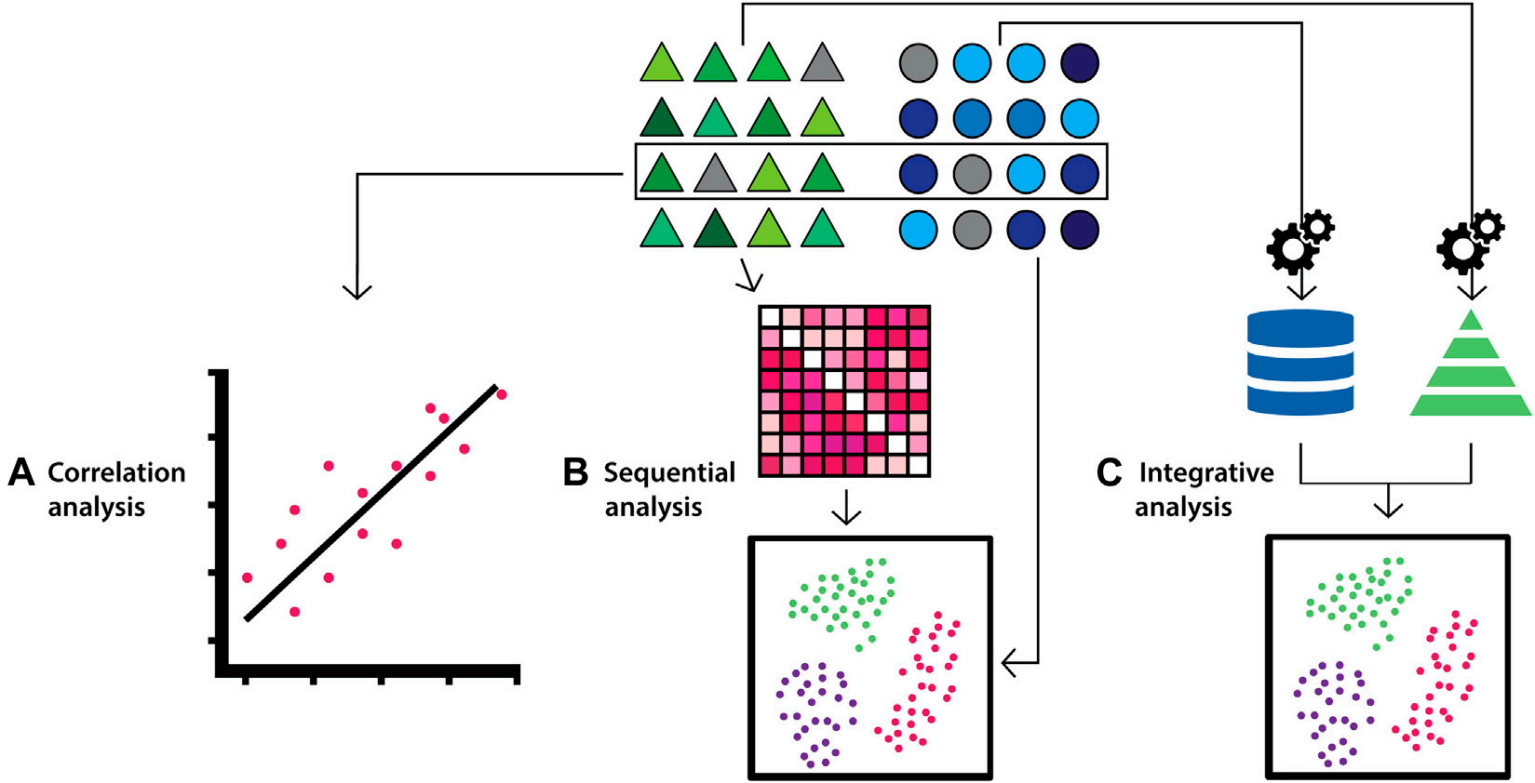
Diagram depicting multi-modal data integration strategies according to the correlation, sequential and integrative categorisations. Triangles (green) and circles (blue) represent datasets from distinct biological data modalities. **(A)** For correlation-based integration strategies, distinct data modalities are processed and analysed independently, and correlations between the data are identified from the results. **(B)** In sequential integration strategies the results of the analysis on one data modality are refined by the integration of additional data modalities in subsequent analyses. **(C)** In the integrative analysis approach, each data modality undergoes feature transformation independently, which are subsequently combined and analysed.

Integrative approaches are commonly used for a range of different studies including classification (*e.g.* disease vs. normal), regression, annotation labelling (*e.g.* based on morphological or phenotypic descriptions), clustering, feature selection (biomarker discovery) and association studies. These studies share some strategies when categorising integrative approaches of multi-modal data. One strategy is to categorise the approaches into correlation analysis where the goal is to find correlations from the result obtained from the analysis of individual data types. Others include sequential analysis, where the analysis of one data type is followed by the integration of another data type), and integrative analysis where integrative analysis of all data types are conducted to obtain an overall determination (Figure 2) (Lee et al., 2020).

**FIGURE 3:**
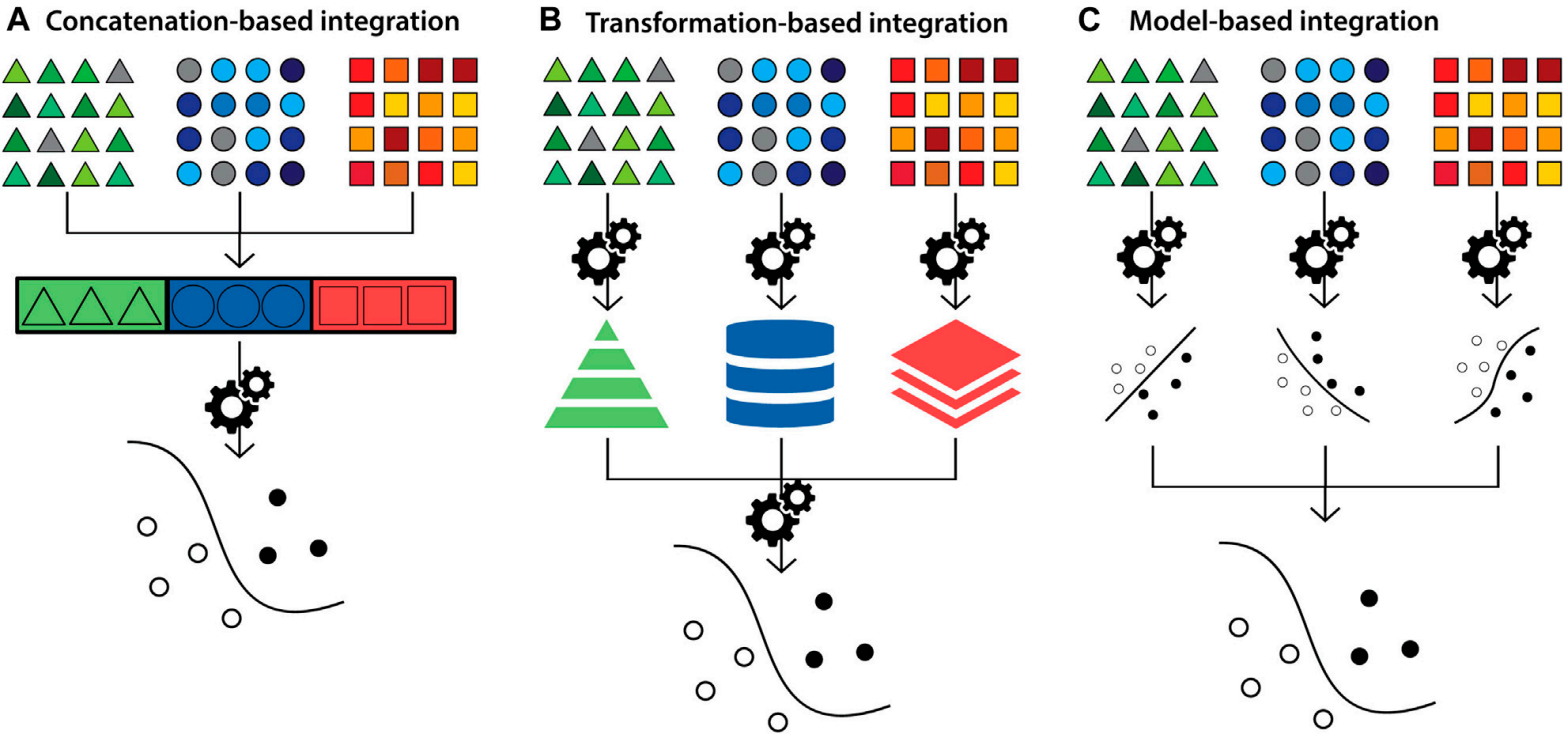
Diagram depicting multi-modal data integration strategies according to the concatenation-, transformation- and model-based categorisation. Triangles (green), circles (blue) and squares (orange) represent datasets from distinct biological data modalities. **(A)** In concatenation-based integration, multi-modal data is joined at the raw or processed level before being passed to an ensuing model for analysis. **(B)** In transformation-based strategies, each data modality undergoes modelling to transform features separately, which are subsequently integrated and passed to a final model for analysis **(C)** In model-based integration, each data modality undergoes modelling and analysis independently, and model outputs are integrated to generate the final result.

Another approach is to classify methods based on the strategies to build a multivariate final model. These methods are classified into concatenation-, transformation- and model-based integration (Figure 3) (Ritchie et al., 2015; Zitnik et al., 2019; Venugopalan et al., 2021). Concatenation-based classification involves combining datasets at the raw or processed level, followed by fitting into a supervised or unsupervised model and then analysis. Depending on the type of the data (*e.g.* images), the data is converted into a feature vector to be combined with other datasets. In transformation-based integration, the original data is transformed separately, and the modelling approach is applied at the level of the transformed matrices *i.e.* data types are integrated during the learning process. Model-based integration involves fitting separate models for individual data types and then combining their outputs to generate knowledge about the overall trait of interest (Ritchie et al., 2015; Venugopalan et al., 2021). The strengths and limitations of integration methods according to this classification strategy, and corresponding examples, are summarised in Supplementary Table S1. These methods are also referred to as early, intermediate and late integration, respectively (Li et al., 2016; Venugopalan et al., 2021).

**FIGURE 4:**
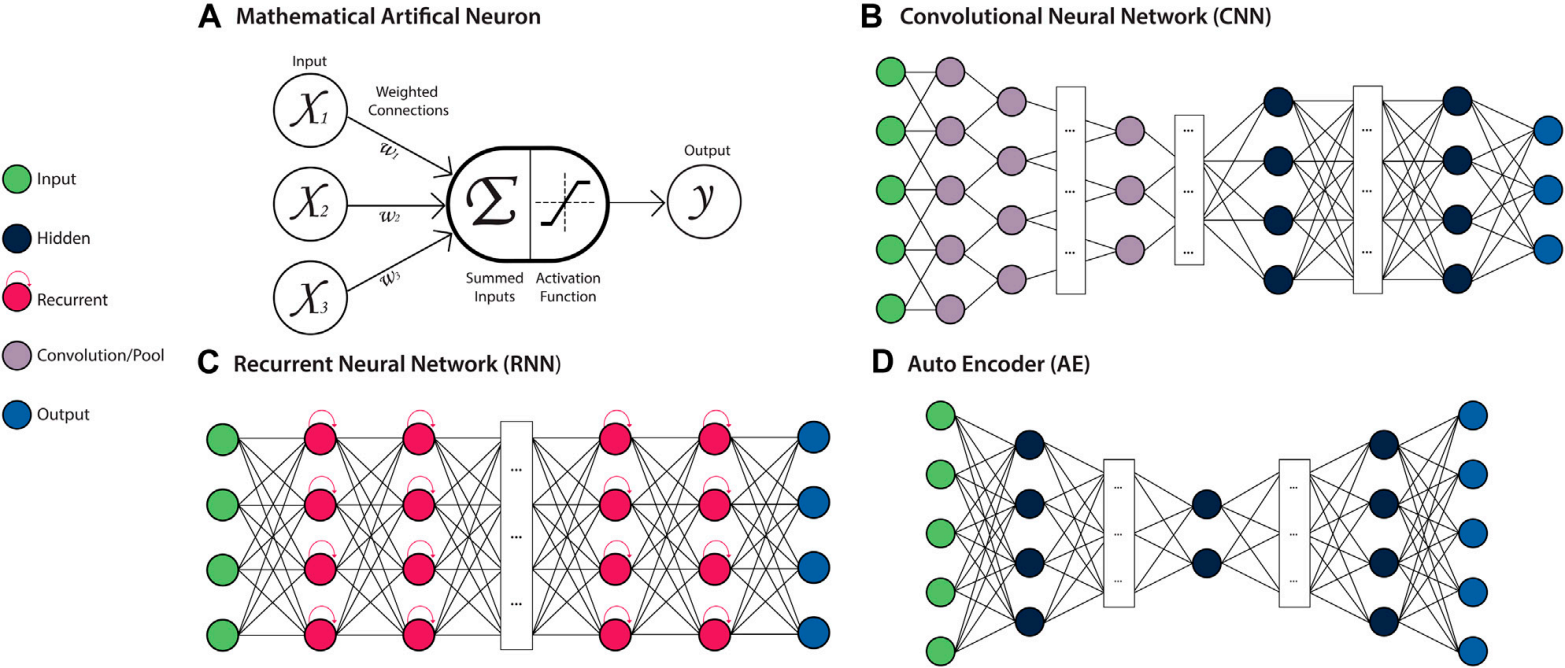
Deep Artificial Neural Network (ANN) Architectures. Left: a key for several types of neurons used in ANN architectures. **(A)** Mathematical model of a neuron. The weighted (Wi) sum of all inputs (Xi) to the neuron is computed and passed to the activation function, which produces the neurons output. This output is propagated as an input to neurons in subsequent layers of the network. **(B)** A Convolutional Neural Network (CNN) is a feed-forward ANN architecture containing convolutional and pooling layers, which allow local patterns to be learned and detected in a spatially invariant manner. **(C)** A Recurrent Neural Network (RNN) is a recursive ANN architecture containing neurons with an internal memory state, which retain information about prior inputs to the model. **(D)** An Autoencoder (AE) is a feed-forward ANN architecture that is comprised of an encoder module that learns a latent representation of the input, and a decoder module that reconstructs the original input data from the encoded representation.

Here we review the commonly-used methods for integrating images with omics data.

### Annotation

The first approach for integrating images with multi-omics data is to derive phenotypic information from imaging data, which is then utilised as annotations to aid in the interpretation of omics data. Thul, Åkesson (Thul et al., 2017) created an image-based map of the human subcellular proteome. They integrated transcriptomics data with high-resolution immunofluorescence microscopy images to determine the subcellular location of 12,003 proteins in various cell lines. Traditional image-derived annotations are usually manually curated in the form of morphological, biochemical, or physiological descriptions or measurements. Moreover, this information is also used for categorical classifications (*e.g.* presence or absence of a specific phenotype) (Hériché et al., 2019). For instance, in the context of cellular senescence, microscopy images of cells stained for senescence-specific markers such as senescence-associated beta-galactosidase (SA-β-gal) are often used to determine the presence of the senescent phenotype (Dimri et al., 1995). This determination can be further supported through quantifying the expression levels of proteins relevant to the senescent phenotype, such as cell-cycle arrest markers p21 and p16. Moreover, staining of the cellular membranes, cytoskeleton or cytoplasm provides morphological information. Cells present with a distinct morphology after transitioning into the senescent state, including enlarged and irregular cell shape, increased granularity and multinucleation (Biran et al., 2017).

Although traditional joint-analyses of multi-modal data are informative and relatively accurate, they are limited in their ability to identify patterns in complex biological data that often contain thousands of features. Therefore, features most relevant to each data type must first be identified and extracted from the raw data before they can be integrated and analysed to draw biologically meaningful conclusions from them. However, as datasets increase in volume, dimensionality and heterogeneity, our ability to identify and extract meaningful features becomes increasingly difficult and inefficient. This problem can be circumvented using more complex mathematical methods for multi-modal data representation and machine learning (ML) models to integrate multi-modal data.

### Higher-Order Data Representation

The volume and complexity of data derived from images and multi-omics data brings the challenge of joining these data in an integrative framework (Hériché et al., 2019). Multi-modal integration methods look for patterns within and across data types, with or without prior knowledge (supervised or unsupervised) of the identity or label of the samples. Multiple high dimensional data can be incorporated and represented as higher order data structures or tensors (Chollet, 2018). Tensors then undergo dimensionality reduction to be integrated and jointly analysed (Li et al., 2016; Hériché et al., 2019). In the context of multimodal data integration, higher-order data representation and tensor factorisation methods have been used in the biological domain. For instance, Zhang, Liu (Zhang et al., 2012) used simultaneous non-negative matrix factorisation to integrate multi-omics cancer data. Argelaguet, Velten (Argelaguet et al., 2018) performed an integrative analysis of various biological data (drug response, mutation status, and transcriptome and DNA methylation profiles) using a joint matrix factorization approach formulated in a Bayesian framework. Last but not least, Acar, Papalexakis (Acar et al., 2014) performed a joint analysis of nuclear magnetic resonance and liquid chromatography–MS data using tensor factorisation.

### Feature Vector Extraction

Both images and multi-omics data can be represented as numerical descriptors in the form of feature vectors (Hériché et al., 2019). Due to the high dimensional nature of the images and multi-omics data, it is often challenging to combine their respective features in the original input space. Thus, new features from each data type can be extracted and then combined. Depending on the nature of the data, feature extraction methods such as matrix factorisation methods (*e.g.* PCA and NMF) or dimensionality reduction methods like autoencoders are applied (Li et al., 2016; Hériché et al., 2019). This is then followed by the classification or clustering on the combined features. The new features in the lower dimensional feature space are commonly numeric, providing a quantifiable measure of heterogeneity in each data mode and easy integration of their respective features. Feature vectors also provide a more efficient downstream analysis due to their reduced dimensionally (Li et al., 2016). Moreover, they can easily be incorporated into relational data (where the similarity between samples are known) by kernel feature extraction methods (Li and Ngom, 2014; Li et al., 2016).

### Artificial Neural Networks

Artificial Neural Networks (ANN) are a class of ML algorithms that are based on many processing units (or “neurons”), typically organised into multiple layers which are inter-connected via edges to form a network (Figure 4A). These edges are assigned weights, which determine the strength of the connection between neurons and are adjusted throughout the network’s learning process to improve the model performance. The neurons of a network’s *input layer* contain the initialising data, which undergoes some transformation at the neurons of one or more *hidden layers*, followed by an *output layer* which produces the final result. The neurons contained within the hidden layers compute the weighted sum of their inputs, apply an activation function, and produce the output (Angermueller et al., 2016b). The activation functions of neurons within hidden layers are typically non-linear, allowing inputs to be transformed in a manner that simultaneously increases the selectivity and invariance of the data representations (features) (LeCun et al., 2015). In “Deep” ANNs containing multiple hidden layers, the outputs from one layer act as input to the following layer. The compounding non-linearity allows for features of increasing complexity to be learned in a hierarchical manner as information progresses through the network. These features are optimised according to the specific task for which the model has been trained, typically classification, regression, or recognition (LeCun et al., 2015). As biological systems are inherently non-linear, this ability to generate intricate nonlinear input-output mappings is of great benefit for resolving the heterogeneity and complexity contained within biological data (Willy et al., 2003; Janson, 2012). The features learned by ANNs can also be extracted as feature vectors from the intermediate layers of the trained model, and subsequently combined for downstream integrative analyses (Chen et al., 2020). By utilising different layer types, neuron connections, activation functions, and learning rules, ANN architectures can be designed with a range of distinct behaviours and applications.

### Convolutional Neural Networks

Convolutional Neural Networks (CNNs) are a feed-forward ANN architecture designed to process input data in the form of multiple arrays (*i.e.* a tensor), making them particularly well-suited to processing raw image data, which usually takes the form of several two-dimensional arrays, representing each colour channel. They are also capable of processing sequence or signal data in the form of multiple one-dimensional arrays. CNNs are typically composed of multiple blocks of convolution and pooling layers which perform the feature learning task (Figure 4B). The convolutional layers contained within CNNs use arrays of weights (kernels) with a pre-defined shape to learn locally distinct patterns in the data through convolution operations. These patterns may represent edges or curves that form an object in an image, or a series of specific bases that form a transcription factor binding site in a genome sequence. The kernels are applied across the entirety of the data array, allowing these features to be detected in a spatially-invariant manner. Pooling layers perform down-sampling operations to merge semantically similar features, leading to robust feature detection and reduced model parameters (LeCun et al., 2015). The final layers of the CNN are fully-connected layers, where neurons are connected to every neuron in the previous layer, which map the learned features to the final output prediction. A more detailed explanation of CNNs can be found in several recent reviews (Gu et al., 2018; Khan et al., 2020).

### Recurrent Neural Networks

Recurrent Neural Networks (RNNs) are a class of ANNs that are specialised for sequential data, such as DNA sequences or time series measurements. RNNs take a single element (*e.g.* an amino acid in a protein sequence) as input at a time, allowing them to process sequences of variable length. The output generated by the neurons of the hidden layer for each element can then be passed as input to another neuron or looped directly back into that same neuron (Figure 4C). This cyclic processing allows the RNN to retain information pertaining to previous outputs in an internal ‘memory’, which is incorporated in the processing of the next element of the sequence. Accordingly, during each new cycle, the output of the hidden layer neurons is generated on the basis of both the new sequence element and the memory of previous sequence elements. As the memory capacity of the basic RNN architecture is relatively limited, a number of derivatives that have been developed to overcome this, including Long Short-Term Memory (LSTM) and Gated Recurrent Unit (GRU) architectures. These architectures are explored further in other reviews (Jozefowicz et al., 2015; Yu et al., 2019).

### Autoencoder

One of the most popular ANN architectures applied for feature extraction is the Autoencoder (AE), which learns features in an unsupervised or ‘self-supervised’ manner. The task of an AE is to encode the input data into an internal representation through learning combinations of non-linear features, and then reconstruct the output from this encoding (Figure 4D). Through iteration, the AE model aims to find a codification of the data that enables the most accurate data reconstruction. This encoding can then be extracted to create a feature vector. AEs have been adapted to suit different data modalities through the incorporation of other ANN architectures, including convolutional AEs for multi-array data and LSTM autoencoders for sequence data (Charte et al., 2018). Various forms of regularisation can also be introduced to ensure the AE is learning a suitably meaningful encoding of the data, as is the case for sparse, denoising and contractive AEs (Zhai et al., 2018). Variational autoencoders (VAEs) are a class of AEs which aim to approximate the underlying distribution of the input data through implementing a variational Bayesian inference approach to encoding (Charte et al., 2018). The generative nature of VAEs make them particularly applicable to multi-modal data integration tasks (Simidjievski et al., 2019). AEs are covered in more detail in a number of recent reviews (Charte et al., 2018; Pulgar et al., 2020).

### Transfer Learning

Transfer learning is the strategy of utilising knowledge learned by a previously trained ANN to enhance the performance of a new model with a different target domain or task. This approach is commonly applied for feature extraction, as ANNs trained on extremely large and diverse datasets tend to learn generic but high-quality features that are transferable across a variety of domain tasks (Pan and Yang, 2010). A number of high-performance models pre-trained on the ImageNet challenge dataset, consisting of 1.4 million images across 10,000 classes, have been utilised for feature extraction from biological imaging data with particular success (Russakovsky et al., 2015). For example, Khan *et al.* (Khan et al., 2019) extracted generic features from breast cytology images using three pre-trained CNNs (GoogleNet (Szegedy et al., 2015), VGGNet (Simonyan and Zisserman, 2014), ResNet (He et al., 2016)), which enabled the detection and classification of malignant cells with an accuracy greater than 97% when combined.

These are but some of the ANN architectures most commonly utilised in multi-modal biological data integration studies. For an extensive review of ANNs and their biological applications, please refer to (LeCun et al., 2015; Angermueller et al., 2016b; Jones et al., 2017; Khamparia and Singh, 2019; Li Y et al., 2019; Tang et al., 2019; Emmert-Streib et al., 2020; Mahmud et al., 2021). Adaptations of many ANN architectures, including CNN, RNN and AE, that are designed to receive graph structured biological data such as gene regulatory networks as input are also available (Jin et al., 2021; Muzio et al., 2021).

## Case Studies

An attractive feature of AI is the ability to identify and extract informative patterns from complex, nonlinear data. Without the need for prior knowledge, AI unveils the mechanism underlying a complex biological process. Recently, ML and deep learning (DL) techniques have been developed and applied in many biomedical health and pharmaceutical-related fields (Gawehn et al., 2016; Mamoshina et al., 2016; Lenselink et al., 2017). These include, prediction of organic chemistry reactions (Wei et al., 2016), optimisation of chemical synthesis (Segler et al., 2018), prediction of pharmacological properties of drugs and drug repurposing (Aliper et al., 2016), modelling structural features of RNA-binding protein targets (Zhang et al., 2015), analysis of drug-induced liver injury (Xu et al., 2015), or the study of human long non-coding RNAs (Fan et al., 2015).

In the context of integrative analysis, depending on the nature of the task (classification, prediction, annotation, or marker discovery), the data types and the amount of data to handle, the constructed models from different ML algorithms are integrated into a single framework to capture the complex mechanism of biological systems. These frameworks are built based on different approaches and as such have different costs and benefits. Network-based fusion methods are able to infer direct or indirect associations in heterogeneous networks. Bayesian-based methods use prior information and model measurements in building the final model. Tree-based models make the final decision based on the trees constructed from individual or collective data types. Additionally, there is a range of deep ANNs that are used to integrate multi-modal data in a single framework (Bersanelli et al., 2016; Li et al., 2016). Here we discuss case-studies that have implemented commonly used frameworks for multi-modal data integration.

Kim *et al.* (Kim et al., 2013) used grammatical evolution neural network (GENN) to predict clinical outcomes for cancer patients by integrating gene copy number, DNA methylation, miRNA and gene expression data. Their computational platform ATHENA allows users to input multimodal omics data. In the first step, the noise variable from each genomic data is filtered out. Individual datasets then go through GENN modelling; the variables that best describe each genomic dataset are selected for the final GENN modelling and integration. An advantage of this framework is its ability to model complex and non-linear relationships between variables, thus identifying interactions that influence variance in an outcome of interest. The final integrated model provides a global view of interaction within and between different levels of genomic data. They tested the final integrated framework on ovarian cancer data from the Cancer Genome Atlas and found that the identified interactions between multiple levels of genomics data are associated with an improved prognosis for ovarian cancer patients.

Chaudhary et al. (2018) used concatenation and DL to integrate mRNA expression, miRNA expression and DNA methylation data to improve clinical outcomes for patients with hepatocellular carcinoma. They implemented an AE model with three hidden layers. For each of the transformed features produced by the AE, they selected survival-associated features through a univariate Cox proportional hazards model. Next, they used these reduced new features to cluster the samples using the K-means clustering algorithm which led to the discovery of two subtypes with significant differences in survival. Furthermore, they validated these two subtypes in five independent cohorts which have an miRNA or mRNA or DNA methylation dataset.

In the context of single cell data integration, Tao et al. (2021) proposed a flexible framework, GLUER, for integrating single-cell omics and imaging data. After normalising the data for each modality, they employ a joint nonnegative matrix factorization (NMF) to identify common factor across data sets of different modalities while maintaining their biological differences. This is followed by using a mutual nearest neighbour (MNN) algorithm to map many-to-many relationships among cells across the data sets, generating factor loading matrices (dimensionality reduced matrices) for each data modality. One factor loading matrix is defined as a reference and the rest as query matrices. A distance between reference and query matrices is computed and used to determine the putative cell pairs between the two datasets. Finally they implement a CNN to learn nonlinear relationships between the factor loading matrices of reference and query datasets. The learnt functions are then used to co-embed the data by combining the reference factor loading matrix and query factor loading matrices.

Yang, Belyaeva (Yang et al., 2021) used AEs to integrate different single cell-sequencing modalities coupled with single cell-imaging data. Their study focused on identifying heterogonous cell states in human naïve CD4^+^ T-cells. In their framework, a different AE model is used to embed each of the data modalities into a shared latent space. The alignment and integration of each embedding within the latent space was performed using an adversarial training approach. Unlike other integration methods (Gundersen et al., 2020), this approach does not require paired data.

Stuart et al. (2019) used canonical correlation analysis (CCA) and MNN to develop a framework for reference assembly and transfer learning for transcriptomic, epigenomic, proteomic, and spatially-resolved single-cell data. First, they used CCA to jointly reduce the dimensionality of the reference and query datasets. These datasets originate from separate single cell experiments but share cells from similar biological states. This is followed by identifying anchors (biologically-matched cells in a pair of datasets) using MNNs in the shared lower-dimensional space. Anchors encode the cellular relationships across datasets that will form the basis for all subsequent integration analyses. A score is assigned to each anchor pair based on the consistency of anchors across the neighbourhood structure of each dataset. Anchors and their score are then utilised to compute “correction” vectors for each query cell, transforming its expression so it can be jointly analysed as part of an integrated reference.

## Applications of Single-Cell and Integrative Multi-Modal Data Analysis in Ageing Studies and Related Resources

Because ageing is a complex biological process, we have selected this particular area of biomedical and health, to showcase studies where multi-modal integration of data has had impact. The complexity of identifying regulators in ageing is due to the fact that ageing is influenced by genetic, epigenetic, transcription, metabolic and post-translation modifications. Systems-level multi-dimensional strategies are therefore required to capture the heterogeneity associated with the ageing phenotype. Due to their improved resolution and advancement, single-cell technologies allow for generation of largescale multi-modal data, which provides opportunities to integrate these datasets to inform our understanding of the mechanism of ageing and age-related disease.

Single cell multi-omics data have been used to discover novel cell types and cell state during ageing; detect cell population shifts and cell-state changes; identify tissue and cell-type specific genes and features; and identify ageing related genes in less abundant cell types (He et al., 2020). The results from these studies are applied in biomarker discovery, drug target identification, regenerative medicine, gene therapy, immune oncology and immunosenescence (Zhavoronkov et al., 2019). For example, Ma, Sun (Ma et al., 2020) created the first single-cell atlas of ageing and ageing interventions in rats that were subjected to a normal and caloric restriction (CR) diet. They studied the ageing-related changes in cell-type composition, gene expression and core transcription factors across tissues due to CR in young and aged rats. Zhang et al. (2019) utilised single-cell whole-genome sequencing to compare somatic mutations in human B lymphocytes in four age groups (newborn, adult, aged and centenarian). They found that somatic mutations increase from <500 per cell to >3,000 per cell across the human lifespan. For a comprehensive list of single cell omics studies in ageing refer to (He et al., 2020).

Single-cell imaging has been applied extensively to the discovery and characterisation of cell types and cell states associated with ageing and age-associated diseases. For example, Phillip et al. (2017) used a range of single-cell imaging technologies to quantify hundreds of biophysical and biomolecular properties of cells obtained from individuals between 2 and 96 years of age. Based on these measurements they were able to identify key phenotypes associated with cellular ageing, such as reduced motility and increased cytoplasmic stiffness, which they used to develop a biological ageing clock. A number of models based on single-cell imaging data have also been developed for the identification of senescent cells (Oja et al., 2018; Kusumoto et al., 2021; Zhai et al., 2021). Wu et al. (2020) has also demonstrated that cellular morphology obtained from imaging data is predictive of the tumorigenic and metastatic potential of individual cells.

The functional annotation of genes linked to ageing and age-associated diseases has also been achieved via single-cell imaging. For instance, Jiao et al. (2019) performed an image-based genetic screen to construct morphological profiles of 125 genes from loci associated with Type-2 diabetes, adiposity, and insulin resistance. Clustering of these profiles revealed novel protein–protein and gene regulatory interactions relevant to Type-2 diabetes. High-throughput single-cell imaging is routinely applied for the discovery of therapeutic compounds to treat a range of age-associated diseases, including Alzheimer’s disease (Honarnejad et al., 2013), osteoarthritis (Nogueira-Recalde et al., 2019), Hutchinson–Gilford Progeria Syndrome (Kubben et al., 2016) and cancer (Caie et al., 2010; Moffat et al., 2014), as well as therapeutics for biological ageing as a whole (Sarkar et al., 2020), often through targeting cellular senescence (Fuhrmann-Stroissnigg et al., 2017).

Integrative multimodal analyses of biological imaging and omics data are popular in ageing-related research. Venugopalan et al. (2021) used deep AEs and CNNs to extract and integrate features from clinical, genomic and neurological imaging data to classify patients according to the severity of their Alzheimer’s disease stage. They also demonstrated that this multi-modal model outperformed single-modality models for the predictive task. Another common application of integrative analysis in age-associated disease is the integration of tissue-level imaging with genomic or transcriptomic data for the identification and classification of cancer sub-types (Shao et al., 2020; Liu et al., 2021b). Alternatively, Sailem and Bakal (2017) performed a correlation-based integrative analysis of single-cell morphology and bulk transcriptional data, finding that alterations in cell shape promoted breast cancer progression through the modulation of NF-kB. Although these studies have typically been limited to the tissue level, the recent advances in single-cell technologies and computational methods discussed in this review hold great promise for enhancing our understanding of the molecular basis of the biological ageing process. For example, Meyer et al. (2020) recently developed a same-cell pharmacogenomics approach, *fate-seq*, which uses live imaging to predict the drug response of individual cells, that are subsequently isolated and profiled using single-cell RNA-seq. With this technique, they were able to identify the transcriptional profile responsible for modulating cancer-drug resistance.

There are many examples where AI has been successfully applied in longevity medicine research, including biomarker discovery (Putin et al., 2016; Moskalev et al., 2017; Zhavoronkov et al., 2021), using deep learning to predict chronological age (Wang et al., 2017) and analysis of relationships between life-style traits (*e.g.* smoking) and accelerated ageing (Mamoshina et al., 2019). Please refer to the following reviews for a comprehensive overview on applications of AI in biomedicine (Fabris et al., 2017; Ching et al., 2018; Rifaioglu et al., 2018; Tsigelny, 2018) and ageing research (Zhavoronkov et al., 2019; He et al., 2020).

The large amount of data generated in ageing research has been organised and disseminated in various databases. The publicly available databases consist of ageing phenotypes, longevity records, ageing- and senescence-related genes, and factors with lifespan-extending effects. These include Human Aging Genomic Resources (HAGR) containing GenAge, AnAge, GenDR, LongevityMap, DrugAge and CellAge (Tacutu et al., 2017). GenAge is a benchmark database for ageing- and longevity-associated genes. CellAge is a manually curated database of senescence-associated genes and DrugAge contains over 500 ageing-related drugs in model organisms. For more information about HAGR databases refer to (Tacutu et al., 2017). Other ageing-research related databases include Geroprotectors (Moskalev et al., 2015), AgeFactDB (Hühne et al., 2014), the Digital Ageing Atlas (Craig et al., 2015), AGEMAP (Zahn et al., 2007), SeneQuest (https://senequest.net/) by ICSA (International Cell Senescence Association). Last but not least, the Aging Atlas (Consortium, 2020) is a curated biomedical database which comprises of multi-omics datasets (sc-transcriptomics, epigenomics, proteomics and pharmacogenomics) and the tools to analyse and visualise the datasets.

## Conclusion

In this review, we provide an overview of the current single-cell omics and imaging technologies, their respective methods for data analysis and common approaches for multi-modal data integration. While single-cell omics and imaging both represent two broad areas of interest, the intention of this review was not to provide an exhaustive treatment of these topics but instead offer a guide to help navigate the growing landscape of these two areas. We expect that the number of new techniques, data analysis approaches, and opportunities for integrating single-cell omics data with images will continue to grow and mature, and we hope that this review provides a reader, especially one who is a beginner to single cell biology, with enough content to learn about these areas more effectively and easily.

Single-cell omics technologies offer unprecedented opportunities to systematically explore cellular and molecular diversity at a single cell resolution. The data generated through these technologies have had a significant impact in understanding the heterogeneity in a cell population or tissue, leading to discovery of novel cell types, their function and their underlying genetic composition. Single cell imaging technologies capture morphological description of tissues and cells. Through the use of these technologies, we are also able to identify and quantify molecular profiles with single-molecule resolution. Advances in different single cell technologies that allow the capture of multiple features of a cell, in combination with the development of new multi-modal data integration approaches presented in this review are rapidly emerging and beginning to present promising results in different fields of biomedical research.
