# Supplementary material for: Computational Methods for Single-Cell Imaging and Omics Data Integration
Source: Front Mol Biosci. 2022 Jan 17;8:768106. doi: 10.3389/fmolb.2021.768106 (PMC8801747; doi:10.3389/fmolb.2021.768106)
Supplement: Supplementary file 1 [file Table1.docx]

Supplementary Table 1. Summary of integration techniques

| **Integration technique** | **Strengths** | **Weaknesses** | **Examples** |
| --- | --- | --- | --- |
| Concatenation-based | - Once multi-modal data has been concatenated appropriately, downstream analysis may be achieved with relatively simple pipelines, including the use of standard statistical methods (Ritchie et al., 2015). - Enables the consideration and possible discovery of interactions between distinct datatypes (Ritchie et al., 2015; Picard et al., 2021). | - Concatenation of datasets of distinct datatypes, such as continuous, discrete, categorical and imaging data in a meaningful way requires appropriate pre-processing and conversions which can be challenging (Li et al., 2016). - Additional downstream processing is often required to address dataset-driven biases, class-imbalances, and dataset-size differences (Ritchie et al., 2015; Picard et al., 2021). - Resultant matrix often contains inflated complexity, noise and high-dimensionality, introducing additional challenges for downstream analysis (Ritchie et al., 2015; Picard et al., 2021). - Dataset-specific distributions are ignored, potentially resulting in identification of patterns in the features which reflect irrelevant information, such as origin from the same dataset.(Picard et al., 2021) | - Chaudharyl et al. (2018) performed concatenation of processed mRNA, miRNA and DNA methylation data into a single matrix, which was fed into an AE to produce a set of integrated features for downstream analysis. |
| Transformation-based | - Robust to data measured in different forms and on different scales, as separate feature extraction and transformation allows for appropriate methods to be selected for each datatype (Ritchie et al., 2015). - Data-type-specific properties from each data set, such as the rich structural information contained in imaging data, are preserved during integration (Ritchie et al., 2015; Li et al., 2016). - Reduces complexity and dimensionality of the data, providing reduced computational complexity and greater flexibility in selection of downstream methods (Li et al., 2016). - Interactions between features from distinct datatypes can be identified and utilised during inference, classification and clustering (Li et al., 2016). - Samples missing from datatypes do not pose significant problems, and trained models may be used to impute profiles of missing data given the datatypes which are provided (Li et al., 2016). - Certain approaches allow for the use of unpaired data (Yang et al., 2021). - Biases such as size differences between datasets are removed in the transformed representation (Picard et al., 2021). - Regardless of the datasets original form, transformed features are typically numeric, allowing for concatenation and the associated benefits such as standard downstream analyses, if desired (Li et al., 2016). | - Often requires the assumption that different datasets share a common latent space reflecting the underlying biological mechanisms (Picard et al., 2021). - Interactions between datatypes cannot be accounted for during separate feature extraction and transformation procedures, which may influence the ability to identify some interaction effects (Ritchie et al., 2015; Li et al., 2016). - Depending on the transformation and modelling approaches, interpretation of features and their subsequent interactions may be challenging (Ritchie et al., 2015; Li et al., 2016). | - The Peng et al. (2021) GLUER method integrates single-cell omics and imaging data through a multi-step process of 1) Joint nonnegative matrix factorisation, 2) mutual nearest neighbours, 3) CNN, 4) co-embedding of data through combining factor-loading matrices of each modality according to the learned mapping. - Yang et al. (2021) presented a probabilistic framework which uses separate AEs to embed each modality into a shared latent space. The distributions of each dataset are then aligned within the latent space using adversarial training. - Stuart et al. (2019) created a framework for integration of paired multimodal data, whereby CCA followed by L2-normalisation is used to project two datasets into a shared latent space. MNN is applied to identify ‘anchors’. Anchors are scored for their consistency across datasets, from which ‘correction’ vectors can be computed for each cell, transforming its expression so it can be jointly analysed. |
| Model-based | - The most suitable approach if datatypes are too heterogeneous to be integrated through concatenation- or transformation-based approaches without the loss of substantial information (Ritchie et al., 2015). - Does not require paired data. - Allows for greater flexibility in selecting analysis methods, including selection of datatype specific approaches (Li et al., 2016). - Results are highly interpretable (Li et al., 2016). | - Each datatype requires a specific hypothesis and analysis (Ritchie et al., 2015). - As integration only occurs at the decision level, interactions of features between datatypes can only be identified if they express within-datatype effects (Ritchie et al., 2015; Li et al., 2016). - Does not allow for machine learning models to utilise complementary information between datatypes in the learning process (Picard et al., 2021). | - Kim et al. (2013) introduced an integration method in their framework ATHENA. Using Grammatical Evolution to optimise Neural Networks (GENN), optimised ANN prediction models are created for each modality separately. The variables from the best model for each modality are then integrated into a single model which outputs a final prediction. - Wang et al. (2021) developed MOGONET, a multi-modal integration framework for supervised classification tasks. Datasets are independently processed and used to train separate graph convolutional networks. The outputted label predictions are used to construct a single tensor of the cross-modality label correlations, which is forwarded to a specialised ANN to produce the final prediction. |

**References**

Chaudharyl, K., Poirionl, O.B., Lu, L.Q., and Garmire, L.X. (2018). Deep Learning-Based Multi-Omics Integration Robustly Predicts Survival in Liver Cancer. *Clinical Cancer Research* 24(6)**,** 1248-1259. doi: 10.1158/1078-0432.Ccr-17-0853.

Kim, D., Li, R., Dudek, S.M., and Ritchie, M.D. (2013). ATHENA: Identifying interactions between different levels of genomic data associated with cancer clinical outcomes using grammatical evolution neural network. *BioData Min* 6(1)**,** 23. doi: 10.1186/1756-0381-6-23.

Li, Y., Wu, F.-X., and Ngom, A. (2016). A review on machine learning principles for multi-view biological data integration. *Briefings in Bioinformatics*. doi: 10.1093/bib/bbw113.

Peng, T., Chen, G.M., and Tan, K. (2021). GLUER: integrative analysis of single-cell omics and imaging data by deep neural network. *bioRxiv***,** 2021.2001.2025.427845. doi: 10.1101/2021.01.25.427845.

Picard, M., Scott-Boyer, M.-P., Bodein, A., Périn, O., and Droit, A. (2021). Integration strategies of multi-omics data for machine learning analysis. *Computational and Structural Biotechnology Journal* 19**,** 3735-3746. doi: 10.1016/j.csbj.2021.06.030.

Ritchie, M.D., Holzinger, E.R., Li, R., Pendergrass, S.A., and Kim, D. (2015). Methods of integrating data to uncover genotype–phenotype interactions. *Nature Reviews Genetics* 16(2)**,** 85-97. doi: 10.1038/nrg3868.

Stuart, T., Butler, A., Hoffman, P., Hafemeister, C., Papalexi, E., Mauck, W.M., 3rd, et al. (2019). Comprehensive Integration of Single-Cell Data. *Cell* 177(7)**,** 1888-1902.e1821. doi: 10.1016/j.cell.2019.05.031.

Wang, T., Shao, W., Huang, Z., Tang, H., Zhang, J., Ding, Z., et al. (2021). MOGONET integrates multi-omics data using graph convolutional networks allowing patient classification and biomarker identification. *Nature Communications* 12(1). doi: 10.1038/s41467-021-23774-w.

Yang, K.D., Belyaeva, A., Venkatachalapathy, S., Damodaran, K., Katcoff, A., Radhakrishnan, A., et al. (2021). Multi-domain translation between single-cell imaging and sequencing data using autoencoders. *Nature Communications* 12(1). doi: 10.1038/s41467-020-20249-2.
